# Untargeted screening for novel autoantibodies with prognostic value in first-episode psychosis

**DOI:** 10.1038/tp.2017.160

**Published:** 2017-07-25

**Authors:** A Zandian, L Wingård, H Nilsson, E Sjöstedt, D X Johansson, D Just, C Hellström, M Uhlén, J M Schwenk, A Häggmark-Månberg, O Norbeck, B Owe-Larsson, P Nilsson, M A A Persson

**Affiliations:** 1 Affinity Proteomics, SciLifeLab, School of Biotechnology, KTH—Royal Institute of Technology, Stockholm, Sweden; 2 Department of Clinical Neuroscience, Karolinska Institutet at Center for Molecular Medicine, L8:01, Karolinska University Hospital Solna, Stockholm, Sweden; 3 Psykiatri Nordväst, SLSO, Karolinska University Hospital Solna, Stockholm, Sweden; 4 Department of Immunology, SciLifeLab, Genetics and Pathology, Uppsala University, Uppsala, Sweden; 5 Department of Medicine, Karolinska Institutet at Center for Molecular Medicine, L8:01, Karolinska University Hospital Solna, Stockholm, Sweden; 6 Department of Clinical Neuroscience, Centre for Psychiatric Research, Karolinska Institutet, Section of Psychiatry at Karolinska University Hospital Huddinge, Stockholm, Sweden

## Abstract

Immunological and inflammatory reactions have been suggested to have a role in the development of schizophrenia, a hypothesis that has recently been supported by genetic data. The aim of our study was to perform an unbiased search for autoantibodies in patients with a first psychotic episode, and to explore the association between any seroreactivity and the development of a Diagnostic and Statistical Manual of Mental Disorders, fourth edition (DSM-IV) disorder characterized by chronic or relapsing psychotic symptoms. We collected plasma samples from 53 patients when they were treated for their first-episode psychosis, and 41 non-psychotic controls, after which the patients were followed for a mean duration of 7 years. Thirty patients were diagnosed with schizophrenia, delusional disorder, schizoaffective disorder, bipolar disorder or a long-term unspecified nonorganic psychosis during follow-up, whereas 23 patients achieved complete remission. At the end of follow-up, plasma samples were analyzed for IgG reactivity to 2304 fragments of human proteins using a multiplexed affinity proteomic technique. Eight patient samples showed autoreactivity to the N-terminal fragment of the PAGE (P antigen) protein family (PAGE2B/PAGE2/PAGE5), whereas no such autoreactivity was seen among the controls. PAGE autoreactivity was associated with a significantly increased risk of being diagnosed with schizophrenia during follow-up (odds ratio 6.7, relative risk 4.6). An immunohistochemistry analysis using antisera raised against the N-terminal fragment stained an unknown extracellular target in human cortical brain tissue. Our findings suggest that autoreactivity to the N-terminal portion of the PAGE protein family is associated with schizophrenia in a subset of patients with first-episode psychosis.

## Introduction

Psychiatric diagnostics is based almost exclusively on observed behavioral characteristics and self-reporting, with few existing biological correlates that are clinically useful.^1^ The lack of refined diagnostic tools makes it difficult to distinguish clinically relevant subgroups of psychiatric disorders, although such stratification would possibly improve treatment outcomes.

In the case of schizophrenia, it is well established that aberrations in the dopamine system play an important role in the pathogenesis, in addition to a strong underlying genetic component, and stress.^2^ Further, a growing body of literature suggests that immunology has a role in the development of the disease, at least in a subgroup of patients.^3^ Some autoimmune diseases, including diabetes mellitus type 1, Grave’s disease and systemic lupus erythematosus, are more prevalent in patients with schizophrenia compared with the general population.^4^ Others, such as rheumatoid arthritis, are rarer than would be expected.^5^ In addition, both positive and negative associations between human leukocyte antigen polymorphisms and schizophrenia have been reported.^3, 6, 7^

To our knowledge, the first article on autoantibodies in patients with schizophrenia was published in 1937.^8^ Since then, many studies have investigated autoreactivity and its possible association with psychosis. In conclusion, a majority of these studies show higher frequencies of autoantibodies in psychotic patients compared with controls.^7^ Specifically, autoantibodies in serum directed to the N-methyl-D-aspartate receptor and the voltage-gated potassium channel have been reported in patients with first-episode psychosis.^9^ However, a recent review article concluded that autoantibodies towards brain antigens may be equally prevalent in healthy controls.^10^ Hence, it remains unclear whether autoimmunity has an etiopathological role in psychosis, and, if so, in all cases or in a subgroup of patients.

In this study, we utilized a unique collection of thousands of protein fragments generated within the Human Protein Atlas (www.proteinatlas.org), together with protein microarrays, to search for autoantibody reactivity in plasma samples. Analyzing 53 patients with first-episode psychosis and 41 non-psychotic controls, our aims were to explore the association between autoantibodies and psychosis and to study whether any certain autoantibody reactivity was associated with the severity of psychotic symptoms and/or the eventual establishment of any DSM-IV-defined disorder characterized by chronic or relapsing psychotic symptoms. We hypothesized that a subgroup of patients would have a higher level of autoreactivity compared with the non-psychotic controls, and that pronounced autoreactivity would be associated with increased symptom severity and chronification.

## Materials and methods

### Experimental study design

The study was carried out in four steps. First, we performed a cross-sectional study analyzing plasma samples from 53 patients with first-episode psychosis and 41 controls, assessing differences in autoimmune reactivity by using an untargeted protein microarray set-up, and secondly, confirming the results with a full-length protein in an enzyme-linked immunosorbent assay (ELISA) analysis. Thirdly, we conducted a clinical follow-up study including all 53 patients, investigating whether autoimmune reactivity at admission was associated with symptom severity or could predict subsequent development of schizophrenia, delusional disorder, schizoaffective disorder, bipolar disorder or long-term unspecified nonorganic psychosis. Fourthly, we performed a complementary immunohistochemistry analysis using rabbit antisera against the N-terminal and C-terminal ends of the PAGE2B protein in 44 different human tissues.

### Study population

Consecutive patients with first-episode psychosis were recruited from the Department of Psychiatry (Psykiatri Sydväst), Karolinska University Hospital Huddinge, Sweden, between 2002 and 2010. Eligible patients were examined by a senior consultant psychiatrist. To be included in the study, patients had to suffer from a first-episode psychosis based on the consensus of board-certified psychiatrists and have no previous history of psychosis, nor of any severe somatic illness such as diabetes or heart failure. Psychotic symptoms as well as general psychiatric symptoms were evaluated through the Positive and Negative Syndrome Scale (PANSS) for schizophrenia.^11^ In addition, blood samples were drawn and plasma was stored at −70 °C until use.^12^ The large majority of the patients with first-episode psychosis had been treated with antipsychotics for 1–3 weeks at the time of blood drawing. During the same time period, as the patient samples were collected, control subjects were recruited from a medical drop-in center located in the same hospital and open to patients with non-severe injuries. A senior consultant psychiatrist examined the recruited control subjects by thorough clinical interviews, to verify that these had no actual signs of psychosis nor history thereof, after which blood samples were collected and stored at −70 °C until use. All study participants gave informed written consent to participate and the study was approved in 2001 by the local ethics committee in Stockholm, Sweden (reference number: 471/01).

### Follow-up

The included psychosis patients were followed from the date of study inclusion until the end of the study period (April 28, 2012), or death, at which point information on their current mental health status and psychiatric diagnoses determined during follow-up was collected from medical records. Individuals were classified according to whether or not they had been diagnosed with an established DSM-IV disorder characterized by chronic or relapsing psychotic symptoms during follow-up, defined as having a registered diagnosis of schizophrenia, delusional disorder, schizoaffective disorder, bipolar disorder or several subsequent diagnoses of unspecified nonorganic psychosis. At the end of the study period, the stored plasma samples from patients (obtained during first-episode psychosis) and controls were analyzed (see below).

### Experimental procedures

#### Proteomic analysis of plasma samples using protein fragments

The Human Protein Atlas^13^ hosts information about protein expression of the large majority of all human protein-coding genes (www.proteinatlas.org). The database includes protein fragments designed based on bioinformatics analyses of each protein-coding gene, for which one or several regions of 30–120 (average 82) amino acids have been selected because of their low sequence identity to other human proteins. The selected sequences were cloned, expressed, purified and spotted on protein microarrays.^14^ For this study, we used 2304 protein fragments, representing 1812 protein-coding genes, from the Human Protein Atlas for the initial screening for IgG autoantibody reactivity in plasma samples from the included patients and controls. As previously discussed,^15^ protein fragment on the antigen arrays was selected in an untargeted manner in the order they were produced in the Human Protein Atlas project, where they are utilized for the generation of polyclonal rabbit antibodies and used also to validate the specificity and selectivity. They were initially chosen based on their low sequence identity to other human proteins. The number of protein-coding genes per chromosome represented by the antigens on the array correlate with the total number of protein-coding genes per chromosome and could be assumed to represent a small untargeted sample from the human proteome (Supplementary Figure S1). In addition, another 92 protein fragments were selected based on findings from previous in-house profiling efforts and studies on neuroimmunology.^16, 17, 18, 19, 20, 21, 22, 23^

#### Screening for autoantibodies on planar microarrays

The use of planar microarrays for profiling of IgG autoantibody repertoires in each plasma sample was performed in accordance with previous studies.^14, 15, 24^ In brief, each microarray glass slide was created by immobilizing 384 different protein fragments in 21 identical subarrays, enabling the analysis of 21 samples in parallel on each slide. In total, six different collections of microarray slides were utilized, allowing binding analysis of 2304 human protein fragments.

Plasma samples were diluted 1:250 in assay buffer (1x phosphate-buffered saline (PBS) supplemented with 3% w/v bovine serum albumin Cohn fraction V (Saveen Werner, Limhamn, Sweden) and 5% w/v non-fat milk powder (Semper, Sundbyberg, Sweden)), and 60 μl of each diluted sample was applied on the microarray slides for 75 min. After 75 min, the arrays were washed three times in PBST (1x PBS supplemented with 0.1% v/v Tween-20) and a fluorescently labeled detection antibody goat anti-human IgG Alexa 647 (Invitrogen, Carlasbad, CA, USA) diluted 1:60 000 in PBST was applied for another 75 min. Thereafter, the arrays were washed and dried before being scanned with a microarray scanner (G2565BA, Agilent, Santa Clara, CA, USA). The scanned image was analyzed using GenePix Pro 5.1 (Molecular Devices, Sunnyvale, CA, USA).

#### Validation of autoantibodies by suspension bead arrays

On the basis of the results from the screening on planar microarrays, 29 protein fragments were selected for further analysis and validation by suspension bead arrays. An additional set of 26 protein fragments, covering different regions of the same selected protein targets, were included to extend the epitope coverage of the proteins of interest. Further, 92 protein fragments representing proteins that are suggested to be autoantibody targets in previous neuroimmunology studies^16, 17, 18, 19, 20, 21, 22, 23^ or during previous in-house profiling efforts were included in the analysis for explorative purposes.

Each protein fragment was coupled to different color-coded carboxylated beads (MagPlex Microspheres, Luminex, Austin, TX, USA).^15, 24, 25^ The suspension bead array with covalently coupled protein fragments was then transferred to the wells of a microtiter plate, incubated with 50 μl plasma diluted 1:250 in assay buffer (as described above) and supplemented with 160 μg ml^−1^ His6-ABP (fusion tag present in all fragments). The plate was incubated on rotation for 1 h in dark and at room temperature. After 1 h, the plate was washed three times in 100 μl PBST (0.05% v/v), after which 50 μl detection antibody, R-phycoerythrin-conjugated goat anti-human IgG (Moss, Pasadena, MD, USA), at 1 μg ml^−1^, was added to each well and incubated for 30 min. The microtiter plate was subsequently washed three more times, and fluorescent signals were measured using an Lx200 instrument (Luminex).

#### Epitope mapping of antibodies toward PAGE2B

On the basis of our previous attempts of epitope-mapping seroreactivity to PAGE2B (data not shown), 15-mer peptides with one amino-acid lateral shift, covering the amino acids 5–32 of PAGE2B, were designed and obtained (PEPscreen, Sigma-Aldrich, St Louis, MO, USA). The peptides were synthesized with biotin and a six-carbon linker in the N terminus. Coupling of peptides to color-coded carboxylated beads and the epitope-mapping assay were performed as previously described.^26, 27^ In brief, neutravidin (ImmunoPure NeutrAvidin, Fisher Scientific, Hampton, NH, USA) was covalently coupled to color-coded carboxylated beads. The biotinylated peptides were dissolved according to the manufacturer’s instructions and added to the color-coded carboxylated beads. Beads were then stored at 4 °C until use. Upon analysis of the epitopes, all the color-coded carboxylated beads (with neutravidin) were pooled together, and 5 μl were added to plasma samples that had been previously thawed, diluted 1:50 and pre-incubated in assay buffer (PBS supplemented with 5% bovine serum albumin and 10 μg μl^−1^ neutravidin) for 1 h. For the rabbit antisera toward the N-terminal fragment of PAGE2B (HPA045952, www.proteinatlas.org), the dilution of the antibody was 1:3000. After an additional 1 h incubation, the beads were washed three times in 100 μl PBST and incubated with anti-human IgG-PE (H10104, Invitrogen) and anti-rabbit IgG-PE (111-116-144, Jackson ImmunoResearch, West Grove, PA, USA), respectively, for 30 min and thereafter washed three more times and fluorescent signals were measured using a FLEXMAP3D instrument (Luminex).

### Validation by ELISA with full-length PAGE2B protein

#### Vector construction for expression of PAGE2B protein

The full-length complementary DNA clone for PAGE2B was obtained as vector synthetic gene (pCMV6-PAGE2B-MycDDK, Prod No. RC220466, Origene, Rockville, MD, USA) and recloned into the high-expression SFV-replicon vector, DREP-E2A-EGFP.^28, 29^ First, the PAGE2B segment of pCMV6-PAGE2B-MycDDK was PCR-amplified with primers adding the restriction sites *Xma*I and *Spe*I at the 5′ and 3′ ends, respectively. The PCR product and the vector DREP-E2A-EGFP were digested using *Xma*I and *Spe*I, and the resulting fragments purified on agarose gel (GeneJet gel extraction kit, ThermoFisher). The digested PCR product and vector were ligated using Quick Ligation (New England Biolabs, Ipswich, MA, USA), resulting in the vector DREP-E2A-PAGE2B.

In a second step, a double Strep-tag was added by PCR amplification from the plasmid pcScFv(XN)-1:7,^30^ and inserted at the *Xho*I and *Spe*I sites in the DREP-E2A-PAGE2B plasmid. The final vector was called DREP-E2A-PAGE2B-Strep, coding for the peptide sequence of PAGE2B fused to double Strep-tags.

#### Expression and purification of PAGE2B protein

Freestyle HEK 293 cells (Life Technologies, Carlsbad, CA, USA) were grown at 37 °C, 8% CO_2_, 125 r.p.m. shaking. The cells were transfected with the DREP-E2A-PAGE2b-Strep vector using the 293fectin Transfection Reagent (Life Technologies) using the manufacturer's protocol. Forty-eight hours post transfection, cells were harvested by centrifugation, followed by lysis for 40 min at room temperature in 200 μl Cell-lytic M (Sigma-Aldrich) per ml cell suspension. The lysate was spun down 1500 *g* for 20 min and sterilized by 0.4 μm filtration (Merck Millipore, Billerica, MA, USA).

For affinity purification, the filtrate was loaded onto a StrepTrap HP 1 ml column (GE Healthcare, Little Chalfont, UK), and the protein was eluted with 2.5 mM desthiobiotin in PBS. The purity and size of the protein was analyzed and confirmed by gel electrophoresis using the NuPAGE system with pre-cast 4–12% Bis-Tris gels (Life Technologies) in MOPS buffer, followed by Coomassie staining and western blot. In the western blot analysis, two different rabbit antisera raised and purified against the C-terminal fragment of the PAGE2B protein (HPA052619, www.proteinatlas.org) and the N-terminal fragment of the PAGE2B protein (HPA045952, www.proteinatlas.org) were used, and diluted 1:800 in PBST (0.05% v/v). Secondary detection reagents were goat anti-rabbit Fab-AP (Pierce, Waltham, MA, USA) diluted to 2 μg ml^−1^ in PBST (0.05% v/v), or antibody directed toward the Strep-tag, Streptactin-AP (IBA, Göttingen, Germany) diluted 1:1000 in PBST (0.05% v/v).

#### ELISA analysis

The recombinant purified full-length PAGE2B protein was used to establish an ELISA. Each well was coated with 0.6 μg purified protein diluted in 50-μl 0.05 M carbonate–bicarbonate buffer (pH 9.6) and incubated overnight at 4 ^o^C. The plate was washed once with PBST (0.05% v/v) and blocked for 1.5 h with 5% (w/v) bovine serum albumin in PBS, and then washed three times with PBST (0.05% v/v) before incubation with the diluted plasma samples.

Crude plasma samples were centrifuged at 3000 *g* for 5 min and diluted 1:40–1:5120 in eight steps in PBS supplemented with 2.5% (w/v) bovine serum albumin in PBST (0.05% v/v). The diluted plasma samples were added to the wells coated with PAGE2B (as above), and incubated at 4 °C overnight. The next day, three washings with PBST were performed, and alkaline phosphatase-conjugated goat anti-human Fc antibody (2 μg ml^−1^ in PBST, Pierce) added, and the plates incubated for 2 h at room temperature. After three additional washes, 50 μl of the substrate Phos (Microwell Phosphatase Substrate System) was added and signals measured at 620 nm.

### Immunohistochemistry analysis

Expression of the PAGE2B protein was explored in 44 different normal human tissues within the Human Protein Atlas,^31^ using the two rabbit antisera previously described (one raised against the C-terminal fragment of PAGE2B (HPA052619, www.proteinatlas.org) and the other raised against the N-terminal fragment (HPA045952)). Briefly, formalin-fixed and paraffin-embedded tissue material was used, from which regions of interest were selected and collected into tissue microarrays and sectioned 4 μm thin. After baking, the slides were deparaffinized and treated with heat-induced antigen retrieval (pH 6). Subsequently, the immunohistochemical protocol was performed as previously described,^32^ the primary antibody incubated 30 min and the secondary antibody incubated 20 min, followed by 5 min 3,3'-diaminobenzidine. All reagents used for immunohistochemistry were manufactured by ThermoFisher Scientific (Waltham, CA, USA) and Lab Vision (Fremont, CA, USA), and immunohistocehmistry was performed in Autostainer (ThermoFisher Scientific). Mayer’s Hematoxylin was used for counterstaining (Histolab, Västra Frölunda, Sweden), and the results were digitalized with a scanning (Aperio Scanscope XT, Richmond, IL, USA).

### Data analysis

Two different statistical approaches were applied to define the presence of seroreactivity in plasma samples analyzed on planar microarrays. First, each sample was evaluated by its median signal intensity in all targets plus 5, 10, 15 and 20 times the quartile deviation, generating sample-specific cutoffs with varied stringency. Reactivities were then converted to binary signals and reactivity frequencies were compared between samples using Fisher’s exact test. The second approach was based on the comparison of the median-normalized signals for each sample. The signals were log_2_-transformed and compared between samples using the Mann–Whitney *U*-test. Antigens with significantly higher levels of seroreactivity across samples (the *P*-value cutoff set to 0.05 for Fisher’s exact test and 0.01 for the Mann–Whitney *U*-test) were selected for further analysis on suspension bead arrays.

A similar approach was applied for analysis of the suspension bead array data. In this case, we analyzed one well at a time, using the median signal intensity and the quartile deviation of each well as comparison. Significant differences were explored using Fisher’s exact test.

Linear epitopes were characterized from the suspension bead arrays, and the epitope sequences were searched for sequence similarity by BLASTP 2.2.29+ (ref. 33) with default settings (see Supplementary Methods for further details).

In the PAGE2B ELISA assay, the cutoff for positive samples was determined by the background-subtracted signals of the 11 negative control samples (C1–11) plus four times the s.d. at 1:40 dilution. One of the patient samples (P20) that showed a consistently strong reactivity to PAGE2B was used as internal control to normalize between the different plates.

Descriptive statistics was used to summarize characteristics of the included patients and controls. Differences in PANSS scores between patients who subsequently achieved complete remission versus those who were diagnosed with different forms of established DSM-IV disorders characterized by chronic or relapsing psychotic symptoms were analyzed by Welch’s *t*-test (the *P*-value set to 0.01).

Odds ratios as well as relative risks for developing schizophrenia or any of the other DSM-IV disorders listed in Table 1 were calculated in patients with autoreactivity to the PAGE protein family (PAGE2B/PAGE2/PAGE5). The significance level was set to 0.05. All analyses were performed in R (v. 3.3.1), a language and software for statistical computing.^34^

## Results

In this study, we performed an untargeted autoantibody profiling in 53 patients with first-episode psychosis and 41 non-psychotic controls. After assessing autoreactivity at admission, patients were followed for a mean of 7 years with regard to disease progression, allowing us to study the association between autoreactivity at admission and subsequent chronification of symptoms. Plasma autoreactivity was assessed in the following three steps: first, we screened for antibody reactivity toward 2304 protein fragments, representing 1812 human protein-coding genes, by using planar protein microarrays. Second, protein fragments to which the autoantibody reactivity was significantly different in the patient- and control groups were used to generate a suspension bead array, in which the binding to these specific protein fragments was reassessed. Third, ELISA confirmed binding with full-length protein. On the basis of the results, we performed an additional immunohistochemistry analysis of 44 different human tissues.

### Characteristics of study participants and results from follow-up

Clinical results are summarized in Table 1. The mean age at study inclusion was 28 and 33 years for patients and controls, respectively. Men were more prevalent in both groups. The mean follow-up time was 7 years (range 2–12 years). Thirty out of fifty-three patients were diagnosed with a DSM-IV disorder characterized by chronic or relapsing psychotic symptoms during follow-up, of which 14 individuals were diagnosed with schizophrenia. The mean PANSS score at study entry was 65. A subsequent diagnosis of schizophrenia was associated with higher PANSS scores at study entry (Welch’s *t*-test *P*-value<0.01, Table 1). Further, a diagnosis of delusional disorder was associated with a lower score on the Negative Scale at study entry (Welch’s *t*-test *P*-value<0.01, Table 1).

### Results from autoantibody screening

**Figure 1:**
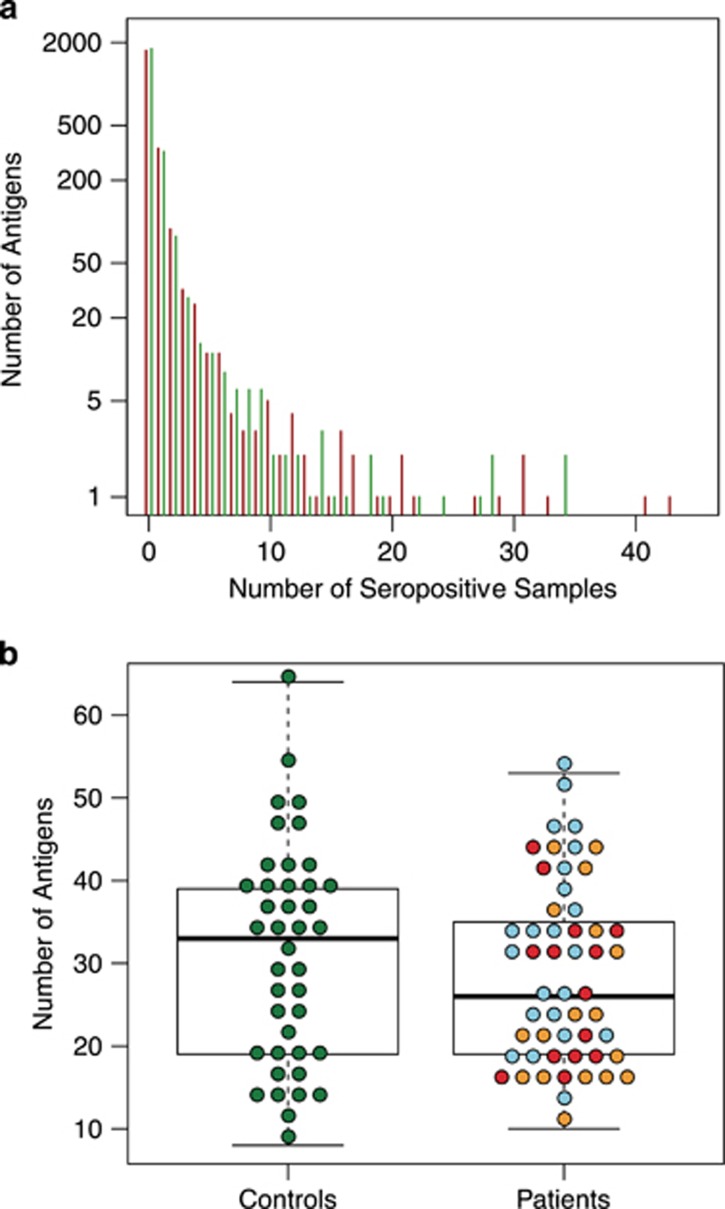
(**a**) General levels of seroreactivity in plasma samples from patients with first-episode psychosis (red bars) and non-psychotic controls (green bars). Results from the untargeted screening with planar microarray assays are shown. The majority of the reactive protein fragments (out of the 2304) were targets for seroreactivity in very few individuals (upper left), whereas a few protein fragments were seropositive in a majority of the samples. (**b**) Distribution of the number of reactive antigens in plasma samples from patients with first-episode psychosis versus non-psychotic controls. Results from the untargeted screening with planar microarray assays are shown. Red dots depict values for patients who subsequently were diagnosed with schizophrenia; yellow dots depict values for patients who developed delusional disorder, schizoaffective disorder, bipolar disorder or unspecified nonorganic psychosis; and blue dots depict values for patients who achieved complete remission.

Autoantibodies were detected in the plasma samples of all study participants. The majority of the 2304 screened protein fragments were targets for seroreactivity in single or very few individuals, whereas a few protein fragments were targets for seroreactivity in almost all plasma samples (Figure 1a). The median level of seropositivity per individual was slightly higher in control subjects compared with patients with first-episode psychosis, although not significantly (Figure 1b).

In the screening on planar microarrays, we identified 29 antigens with differential seroreactivity in patients and controls.

### Results from suspension bead arrays

**Figure 2:**
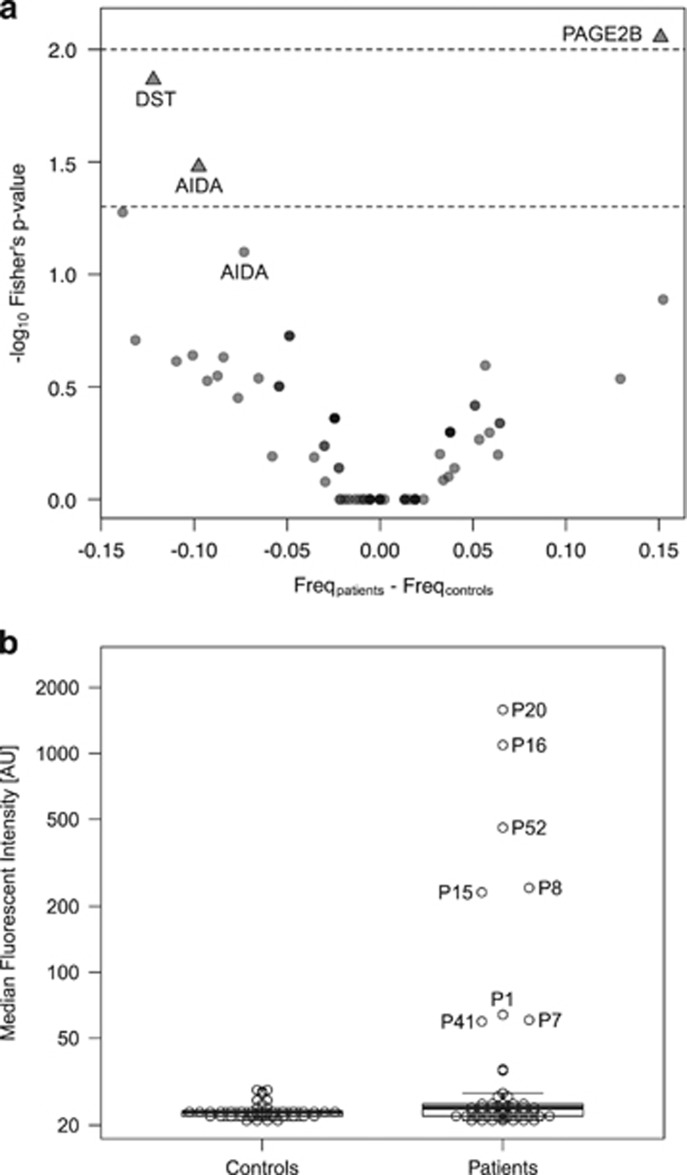
(**a**) Antigens with distinct and differential seroreactivity in patients with first-episode psychosis or non-psychotic controls. Results from the suspension bead array assays summarized in a volcano plot. Seroreactivity against three antigens, the N-terminal fragment of PAGE2B, DST (dystonin) and AIDA (axin interactor, dorsalization associated), was significantly differently distributed in patients versus controls, based on Fisher’s exact test with a cutoff set to five times the quartile deviation of each sample median. Please note that there are two different antigens of AIDA in the figure. (**b**) Plasma IgG seroreactivity to the N-terminal fragment of PAGE2B in patients with first-episode psychosis versus non-psychotic controls. Results from the suspension bead array assays showing higher IgG seroreactivity in patients with first-episode psychosis compared with controls.

Validation by suspension bead array assays confirmed a significantly different pattern of seroreactivity in patients versus controls for three of the 29 antigens (Fisher’s test *P*-value<0.05; Figure 2a). Autoantibodies toward a fragment of the PAGE2B (P antigen family, member 2B) protein, representing its N-terminal region, were observed in eight patients with first-episode psychosis, whereas no control subject presented with seropositivity toward the N-terminal fragment of PAGE2B (Figure 2b). Out of the mentioned eight patients, five were diagnosed with schizophrenia during follow-up, whereas three achieved complete remission. The mean age at admission for first-episode psychosis of the five patients who developed schizophrenia was 23 years. For the two other antigens, DST (dystonin) and AIDA (axin interactor, dorsalization associated), seroreactivity was more prevalent in the non-psychotic controls compared to patients with first-episode psychosis (Figure 2a).

### Sequence alignment of the PAGE2B protein

**Figure 3:**
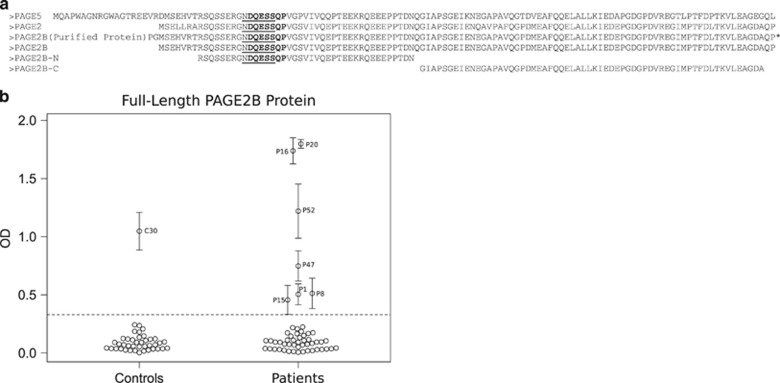
(**a**) Alignment of the N- and C-terminal fragments of PAGE2B to other PAGE protein family members. Alignment of the N- and C-terminal fragments of PAGE2B (PAGE-N and PAGE-C), cloned full-length protein of PAGE2B and the proteins PAGE2 and PAGE5. The epitope-mapped sequence from the patient samples, NDQESS, is underlined and the epitope-mapped sequence from the antisera toward the N-terminal PAGE2B, DQESSQP, is shown in bold. *, EK site with double StrepTags (50 amino acids). (**b**) Plasma IgG seroreactivity to full-length PAGE2B in patients with first-episode psychosis versus non-psychotic controls. Results from the enzyme-linked immunosorbent assay (ELISA) are shown. The error bars represent the s.d. The dashed line is the cutoff value based on the mean intensity of all negative controls+4 × s.d. Please note the similar patterns of seroreactivity in the suspension bead arrays (Figure 2b) and the ELISA.

In an effort to explore potential targets of autoantibodies reactive to the N-terminal fragment of the PAGE2B protein, we compared the sequence of PAGE2B to other human proteins. A high sequence identity to two other human proteins, PAGE2 and PAGE5, was found (Figure 3a). Both the N-terminal and the C-terminal ends of PAGE2B matched PAGE2 and PAGE5 with high sequence identity. In fact, the N-terminal part of PAGE2B, which was the protein fragment used in our proteomic analyses, was found to be identical to PAGE2, and only differed with regard to two amino acids compared with PAGE5.

Furthermore, by epitope-mapping the eight patient samples that were seropositive for the N-terminal fragment of PAGE2B (PAGE-N), we could identify linear epitopes, with the common sequence NDQESS (Figure 3a and Supplementary Figure S2a). Sequence similarity searches from BLASTP resulted in only three matches with perfect identity match (Supplementary Table S1): PAGE2, PAGE2B and PAGE5. In addition, antisera toward the N-terminal region of PAGE2B (HPA045952, www.proteinatlas.org) was epitope-mapped with the minimal epitope DQESSQP (Figure 3a and Supplementary Figure S2b), resulting in very similar results from the BLASTP searches (Supplementary Table S2). Hence, it is important to note that these antibodies are likely also to be directed to PAGE2 and PAGE5.

### Results from confirmatory analyses in ELISA with full-length PAGE2B protein

Full-length protein was successfully expressed and purified (Supplementary Figure S3), and used to establish an ELISA. Seven patients and one control (C30) were seropositive for the full-length PAGE2B protein in the ELISA (Figure 3b). One patient (P47) who had not shown seropositivity to the N-terminal fragment of the PAGE2B protein in the array analyses was seropositive to full-length PAGE2B protein. Further, two patients (P7 and P41) who had been seropositive to the N-terminal fragment of the PAGE2B protein in the previous experiments were negative to full-length PAGE2B protein. The degree of binding correlated well between the suspension bead array assays and the ELISA (Figures 2b and 3b). Notably, the two plasma samples positive to the whole protein, but not to the N-terminal fragment of PAGE2B (P47 and C30), were both positive to the C-terminal fragment of PAGE2B (Supplementary Figure S4). Whereas the pattern of seroreactivity toward the N-terminal fragment of PAGE2B differed significantly between patients and controls, the pattern of seroreactivity toward the C-terminal fragment was comparable in the two groups (data not shown).

### Seroreactivity to the N-terminal portion of the PAGE protein family

**Figure 4:**
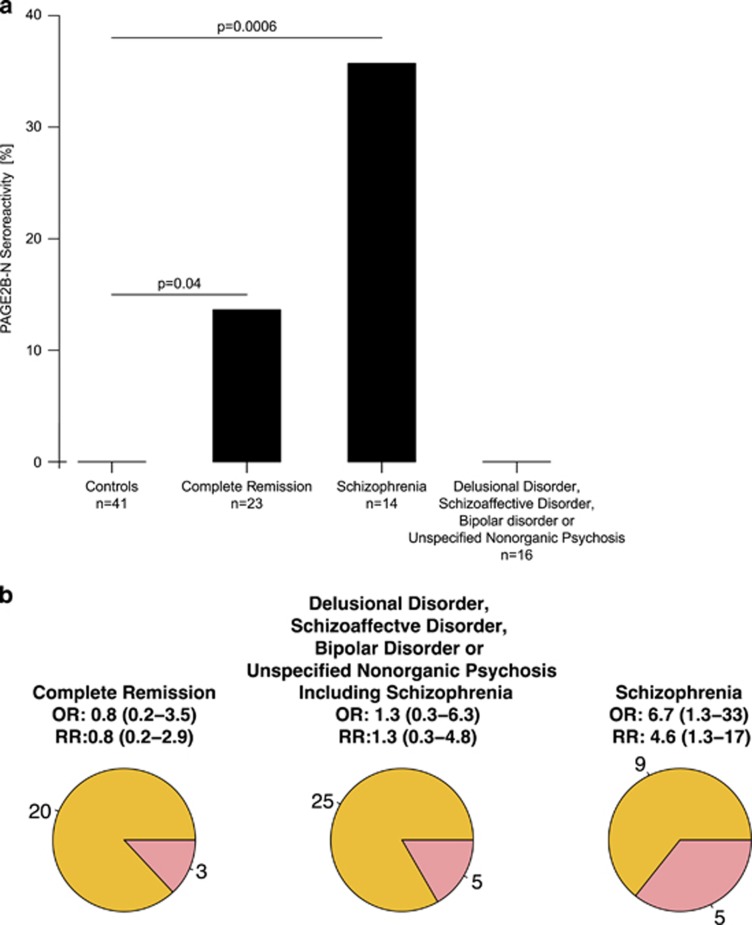
(**a**) Association between seropositivity to the N-terminal fragment of PAGE2B and disease chronification in patients with first-episode psychosis. Controls are included as reference. The proportion frequency of seroreactivity to the N-terminal fragment of PAGE2B in controls, patients who achieved complete remission, patients who were diagnosed with schizophrenia and patients who were diagnosed with non-schizophrenic disorders, respectively. Significant differences between the proportion frequencies were seen in controls versus patients who were diagnosed with schizophrenia (*P*=0.0006) and controls versus patients who achieved complete remission (*P*=0.04). (**b**) Odds ratios and relative risks for complete remission, non-schizophrenic DSM-IV disorders characterized by psychotic symptoms and schizophrenia in patients seroreactive to the N-terminal fragment of PAGE2B during first-episode psychosis. Red sectors of the circle diagrams illustrate the number of seropositive patients and the yellow sectors represent the number of seronegative patients.

Patients with first-episode psychosis who presented with autoantibodies reactive to the N-terminal fragment of PAGE2B (representative for the PAGE2B, PAGE2 and/or PAGE5 proteins) at study inclusion had an increased risk of being diagnosed with a DSM-IV disorder characterized by chronic or relapsing psychotic symptoms (odds ratio (OR) 1.33), in particular schizophrenia (OR 6.7, relative risk 4.6; Figures 4a and b).

### Results from the immunohistochemistry analysis

**Figure 5:**
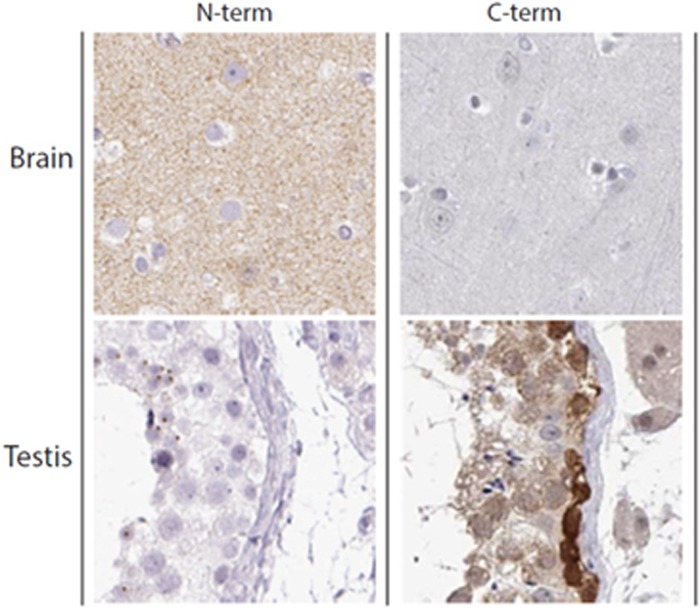
Results from immunohistochemistry analyses with antisera from rabbits immunized with the N- and C-terminal fragments of the PAGE2B protein, respectively. Left panel: the antisera toward the N-terminal fragment of PAGE2B show positive staining for cerebral tissue. Right panel: the antisera towards the C-terminal fragment of PAGE2B show positive staining for testis tissue. Staining of kidney, gastrointestinal tissue and skin were negative for both antisera (not shown).

Polyclonal rabbit antisera raised to the N-terminal fragment of the PAGE2B protein and the C-terminal fragment of the PAGE2B protein, respectively, were applied to a total of 44 different human tissues. We found that the antisera to the N-terminal fragment of PAGE2B mainly were reactive to unknown extracellular structures in the cerebral cortex, whereas antisera to the C-terminal fragment of PAGE2B stained intracellular components of testicular cells (Figure 5).

## Discussion

We have assessed autoantibody repertoires in patients with first-episode psychosis, and performed an untargeted screening toward thousands of autoantigens in this population of patients. We found that all study participants, including all non-psychotic controls, showed some level of seroreactivity toward the 2304 examined protein fragments, representing 1812 human proteins. Although there was a trend toward a higher level of seroreactivity in the control group, with two antigens with significant higher seroreactivity, DST and AIDA, the most significant finding was the antigen represented by the N-terminal portion of the PAGE protein group (PAGE2B/PAGE2/PAGE5), detected in eight patients with first-episode psychosis. Autoantibodies toward this protein were associated with a fourfold increased risk of a future diagnosis of schizophrenia. Immunohistochemistry studies showed that rabbit antisera raised to the N-terminal fragment of the PAGE2B protein mainly were reactive to structures in the human cerebral cortex. Additional epitope-mapping experiments showed that the identified linear epitopes map to the PAGE protein family, and subsequent sequence similarity searches showed highest match to PAGE protein family among the human proteins.

Whereas the global screening for novel autoantibodies has long been hampered by the limited access to broad representations of the proteome, we were able to use the unique collection of protein fragments generated within the Human Protein Atlas to perform an exploratory and unbiased screening for novel autoantibody targets in psychosis. The long and complete clinical follow-up over an average period of 7 years allowed us to draw firm conclusions on disease progression, and to assess the potential prognostic properties of the identified autoantibodies.

There are, however, some important limitations to consider. Even if our cohort of patients with first-episode psychosis has a reasonable size for a pilot study, 53 patients is still a limited number. As previously described,^15^ the different immobilization strategies of the antigens on the different protein microarrays are likely to affect epitope recognition. The use of fragments from human proteins rather than full-length proteins could affect the observed autoantibody reactivities because of the obvious risk of missing out potential interesting epitopes. This could be the reason for why the level of seroreactivity to included protein fragments that represented proteins previously described in the literature as potential autoantigens in schizophrenia^9, 17^ was similar in patients and controls. The lack of stringent matching of patients and controls is another limitation of this study, although the groups had similar age- and sex distributions and were all living in the same metropolitan area of Stockholm, Sweden. Furthermore, as we did not control for socioeconomic status, differences in social and physical environments between patients and controls could have affected the outcome of this study.^35^ The mean age for the patient group, 28 years, can look high, given that many psychotic disorders have their onset in late teens or early 20-ies. We note, however, that the five PAGE-N-seropositive patients who developed schizophrenia had a mean age of 23 years, and a recently cohort study from another Scandinavian country reported that the peak age of schizophrenia onset was between 18 and 31 years, with the upper age limit stretching up to 55.^36^ Still, the mean age at onset in our patient cohort is high, for which we do not have a clear explanation. Lastly, it should be acknowledged that the classification of patients as having achieved complete remission versus having developed a DSM-IV disorder characterized by chronic or relapsing psychotic symptoms, and the subclassification into different diagnostic categories, was made based on diagnoses decided by the attending physician and thus retrieved from medical records, which may have impaired the diagnostic reliability.

In concordance with recent findings indicating that autoantibodies may be equally prevalent in healthy individuals,^10^ we found no significant difference in the general level of seroreactivity in patients versus controls. Indeed, certain antibody specificities occurred primarily in the control group. This finding will warrant further studies of whether some autoantibodies may be 'protective' or associated with better health. However, it can also be seen as a reflection of the overall high degree of individual heterogeneity in human IgG reactivity profiles.^37^

Among the three types of autoantibodies with a significantly different distribution pattern in patients versus controls, autoantibodies toward the N-terminal portion of the PAGE protein family (PAGE2B/PAGE2/PAGE5) were exclusively seen in patients with first-episode psychosis. The subset of patients who were seropositive for the PAGE protein family had a significantly increased risk of being diagnosed with schizophrenia (relative risk 4.6 and OR 6.7). Although based on a relatively small set of patients, this is an intriguing finding, especially considering that this observed association is considerably stronger than most known risk associations for single genetic elements implicated in the development of schizophrenia.^3, 38^

The PAGE protein family is a broad but closely related group of proteins, mainly studied as tumor-associated molecules in the testis, ovaries and mammary glands.^39^ Expression and immunohistochemistry data indicate the presence of PAGE proteins in malignancies in these tissues, but some reports have also suggested a low degree presence of PAGE2B in the human cerebellum and cerebral cortex.^40, 41^ Previous immunohistochemistry analyses on normal tissue have only used rabbit antisera against the C-terminal fragment of PAGE2B, which has stained an intracellular protein of testicular cells, the small intestine and other peripheral tissues (www.proteinatlas.org). Our immunohistochemistry analysis confirmed binding to testicular cells by antisera to the C-terminal fragment of PAGE2B. However, we found that rabbit antisera immunized with the N-terminal fragment of PAGE2B mainly stained unknown extracellular structures in the human cerebral cortex. We consider this an interesting finding, as we performed an unbiased screening for antigens without focusing on antigens of the central nervous system (CNS). However, the data presented in this study are insufficient to claim that the PAGE2B protein is located in the CNS, as there is a possibility that the antisera are staining a different antigen in the CNS, cross-reactive with the N-terminal fragment of PAGE2B.

Autoantibodies to antigens in the CNS or peripheral neurons have been reported in several neurological and psychiatric conditions. However, it is often unknown to what extent these antibodies are the direct cause for induction of symptoms.^42^ In myastenia gravis, autoantibodies have a clear disease-causing effect by attacking the acetylcholine receptor,^43^ whereas in neuromyelitis optica, specific T-cell reactivity is thought to be required for the disease development, in addition to antibodies to aquaporin-4.^44, 45^ Accordingly, putative autoantibodies may be markers for a more general immune reaction rather than representing specific disease-causing agents. Several genetic elements with a role in inflammation and/or immunological reactions have previously been linked to schizophrenia.^3, 6, 38^ Similar to these risk genes, disease-associated autoantibodies may exist in a subgroup of psychotic patients for whom the immunological component has a larger role in the etiopathology than for the remainder of individuals with psychosis. Possibly, this subgroup would benefit from immune-suppressive treatments in addition to the standard neuroleptic regimen. Indeed, clozapine, the neuroleptic drug recommended for patients who fail to respond to standard neuroleptics, has immunosuppressive effects.^46^

In conclusion, we found that a subgroup of patients with first-episode psychosis was seropositive to the N-terminal portion of the PAGE protein family (PAGE2B/PAGE2/PAGE5), and that such seropositivity was associated with the development of schizophrenia. Our findings indicate that these autoantibodies possibly could, if confirmed in larger cohorts, be used as biomarkers for the probability of disease progression in patients with first-episode psychosis, and support the hypothesis that immune aberrations may contribute to the development of schizophrenia in a subgroup of patients. Additional studies are necessary to confirm these findings and to elucidate whether PAGE2B, PAGE2, PAGE5 or another protein cross-reactive to the N-terminal end of PAGE2B is the critical autoantibody target, and to further disentangle its role in the etiopathology of psychosis. We note that affinity proteomic tools can provide novel discoveries and bring important new knowledge in psychiatry.

## Figures and Tables

**Table 1:** Characteristics of patients and controls at admission and data on subsequent disease progression

	*Participants (*N*)*	*Men (*N*)*	*Women (*N*)*	*Age; mean years (s.d.)*^b^	*PANSS*^a^ *scores at admission*			
					*PANSS; mean years (s.d.)*	*Positive; mean years (s.d.) scale*	*Negative scale; mean years (s.d.)*	*GP scale; mean years (s.d.)*
Controls	41	28	13	34 (8.2)	ND			
Patients	53	31	22	28 (8.6)	65 (20)	16 (5.6)	16 (7.0)	33 (10)
								
*Subsequent disease progression and psychiatric diagnoses during follow-up*								
Complete remission	23	10	13	27 (10)	61 (18)	14 (5.1)	15 (5.9)	32 (9.3)
DSM-IV disorders characterized by chronic or relapsing psychotic symptoms	30	21	9	28 (7.6)	68 (21)	17 (5.8)	17 (7.7)	34 (10)
Schizophrenia	14	14	—	27 (7.0)	78 (19)^c^	19 (5.7)	21 (7.3)^c^	38 (10)
Delusional disorder	7	2	5	30 (6.3)	53 (17)	15 (6.5)	11 (2.5)^c^	27 (8.2)
Schizoaffective disorder	3	1	2	31 (12)	48 (12)	11 (4.2)	11 (2.5)	25 (6.0)
Bipolar disorder	3	2	1	21 (4.3)	67 (23)	17 (5.6)	16 (6.9)	34 (11)
Unspecified nonorganic psychosis	3	2	1	32 (11)	73 (25)	17 (3.1)	19 (12)	37 (14)

Abbreviation: PANSS, Positive and Negative Syndrome Scale; GP, general psychopathology; ND, not determined.

a PANSS consisting of three parts: the Positive Scale, the Negative Scale and the General Psychopathology Scale. The mean scores at study inclusion are shown.

b Mean age at study inclusion.

c Significant difference to other psychotic patients at admission (Welch’s *t*-test *P*-value <0.01).
