# Supplementary material for: Untargeted screening for novel autoantibodies with prognostic value in first-episode psychosis
Source: Transl Psychiatry. 2017 Jul 25;7(7):e1177–. doi: 10.1038/tp.2017.160 (PMC5538130; doi:10.1038/tp.2017.160)
Supplement: Supplementary Information [file tp2017160x1.docx]

Supplementary Information

**Table S1. Sequence similarity search using BLASTP.** The identified linear epitope from the autoantibodies in the PAGE2B seropositive plasma samples were mapped to 5 different proteins, but only the PAGE protein family with 100% identity.

**Table S2. Sequence similarity search using BLASTP.** The identified linear minimal epitope of the antisera towards the N-terminal region of PAGE2B (HPA045952), showing the top25 proteins mappings with highest sequence identity with only the PAGE protein family with 100% identity.

**Figure S1.** **Correlation of protein coding genes per chromosome.** The total number of protein coding genes per chromosome, represented by the 2304 antigens on the antigen array (y-axis), correlated with the total number of coding genes by chromosome (x-axis), Spearman’s rho = 0.85, p-value = 1.3 x 10^-7^. Therefore, the 2304 antigens can be assumed to represent a small, untargeted sample from the human proteome.

**Figure S2.** **Epitope mapping the PAGE2B reactivities**. Eight samples that showed seropositivity towards PAGE2B were epitope mapped on peptides covering the N-terminal region of PAGE2B, and a linear stretch of amino acids, NDQESS, was found to show reactivity on both epitope regions (a). Binding was only observed towards this sequence when presented either to the N-terminal or C-terminal end of the peptide, suggesting potential sensitivity for steric hindrance when the sequence is positioned in the middle of the peptide. The antisera (HPA045952) towards the N-terminal region of PAGE2B was also epitope mapped and found to have a minimal epitope with the amino acids DQESSQP.

**Figure S3. Analyses of purified full-length PAGE2B protein.** *Panel A:* Commassie staining of purified PAGE2B sample quantified against BSA standard.  *Panel B:* Western blot showing reactivity against Streptactin-AP. *Panel C:* Western blot showing reactivity to two different monospecific rabbit-antisera raised against the N-terminal region of PAGE2B (antisera HPA045952, [www.proteinatlas.org](http://www.proteinatlas.org)) and antisera raised against the C-terminal region of PAGE2B (antisera HPA052619).  Please note that in all panels the highest stained protein fraction has an approximate molecular weight of 33 kD, which likely corresponds to a dimer of PAGE2B (predicted monomer MW 16.7 kD), which has also been suggested by Gjerstorff and co-workers when expressing a related GAGE protein^1^.

**Figure S4. Correlation of plasma IgG reactivity against PAGE2B-N and PAGE2B-C terminal fragments vs. full-length PAGE2B protein.** IgG binding to fragments were measured in the suspension bead array set up (y-axis), and reactivity against the whole protein in ELISA (x-axis).

Table S1.

| **UniProt ID** | **Gene** | **E-value** | **Score** | **Identity [%]** |
| --- | --- | --- | --- | --- |
| Q96GU1-2 | PAGE5 | 16 | 44 | 100 |
| Q7Z2X7 | PAGE2 | 16 | 44 | 100 |
| Q5JRK9 | PAGE2B | 16 | 44 | 100 |
| Q96GU1 | PAGE5 | 17 | 44 | 100 |
| Q13127-4 | REST | 150 | 37 | 83.3 |
| Q13127 | REST | 150 | 37 | 83.3 |
| O60307 | MAST3 | 150 | 37 | 83.3 |

Table S2.

| **UniProt ID** | **Gene** | **E-Value** | **Score** | **Identity** |
| --- | --- | --- | --- | --- |
| Q96GU1-2 | PAGE5 | 1.5 | 52 | 100 |
| Q7Z2X7 | PAGE2 | 1.5 | 52 | 100 |
| Q5JRK9 | PAGE2B | 1.5 | 52 | 100 |
| Q96GU1 | PAGE5 | 1.5 | 52 | 100 |
| Q12888 | TP53B | 67 | 40 | 71.4 |
| Q12888-3 | TP53B | 67 | 40 | 71.4 |
| Q12888-2 | TP53B | 67 | 40 | 71.4 |
| Q8NHQ1-3 | CEP70 | 120 | 38 | 83.3 |
| Q96JE7-3 | SC16B | 120 | 38 | 83.3 |
| Q8NHQ1-2 | CEP70 | 120 | 38 | 83.3 |
| Q8NHQ1 | CEP70 | 120 | 38 | 83.3 |
| Q02447-5 | SP3 | 120 | 38 | 83.3 |
| Q02447-6 | SP3 | 120 | 38 | 83.3 |
| Q02447-2 | SP3 | 120 | 38 | 83.3 |
| Q02447 | SP3 | 120 | 38 | 83.3 |
| P46087-2 | NOP2 | 120 | 38 | 83.3 |
| P46087 | NOP2 | 120 | 38 | 83.3 |
| P46087-4 | NOP2 | 120 | 38 | 83.3 |
| Q96JE7 | SC16B | 120 | 38 | 83.3 |
| P51587 | BRCA2 | 120 | 38 | 83.3 |
| Q86VP3-4 | PASC2 | 170 | 37 | 83.3 |
| Q86VP3 | PASC2 | 170 | 37 | 83.3 |
| Q86VP3-3 | PASC2 | 170 | 37 | 83.3 |
| Q86VP3-2 | PASC2 | 170 | 37 | 83.3 |

Figure S1.

Figure S2.

**a**

**b**

Figure S3.


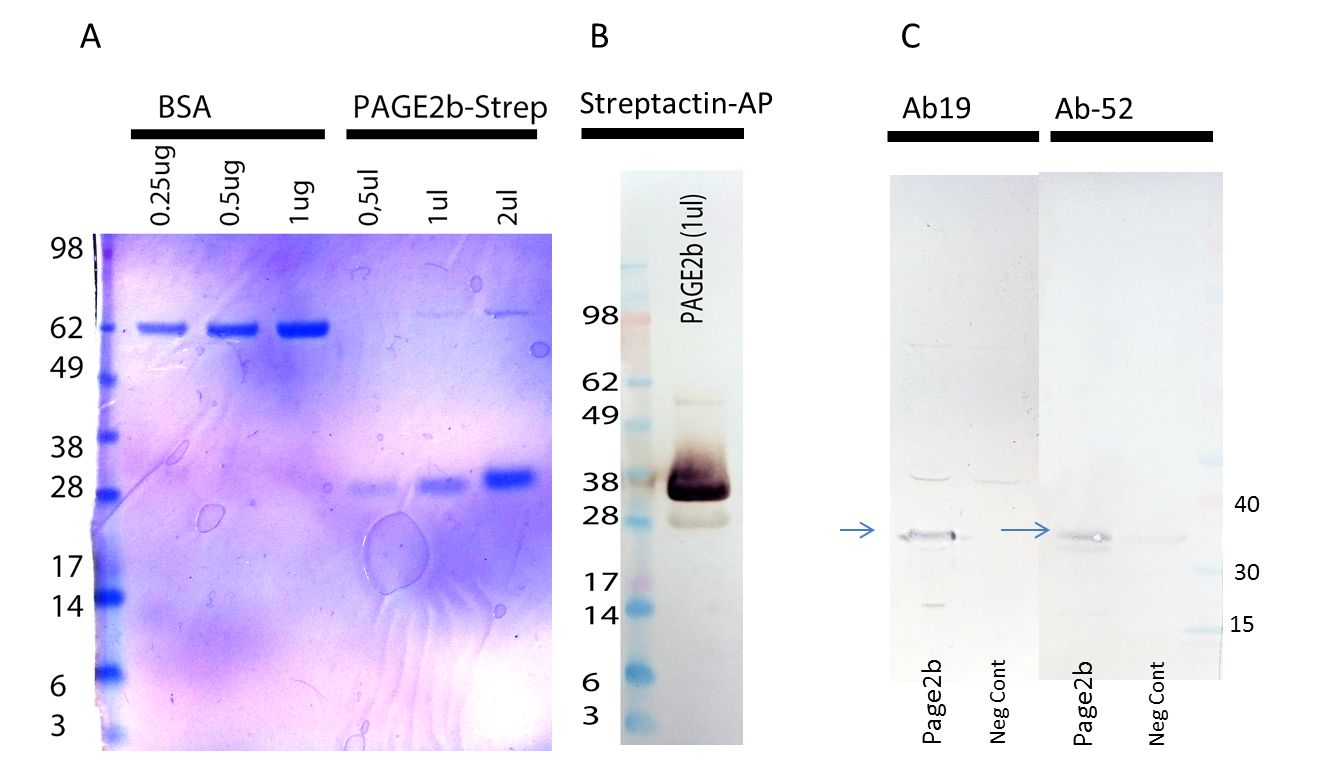


Figure S4.


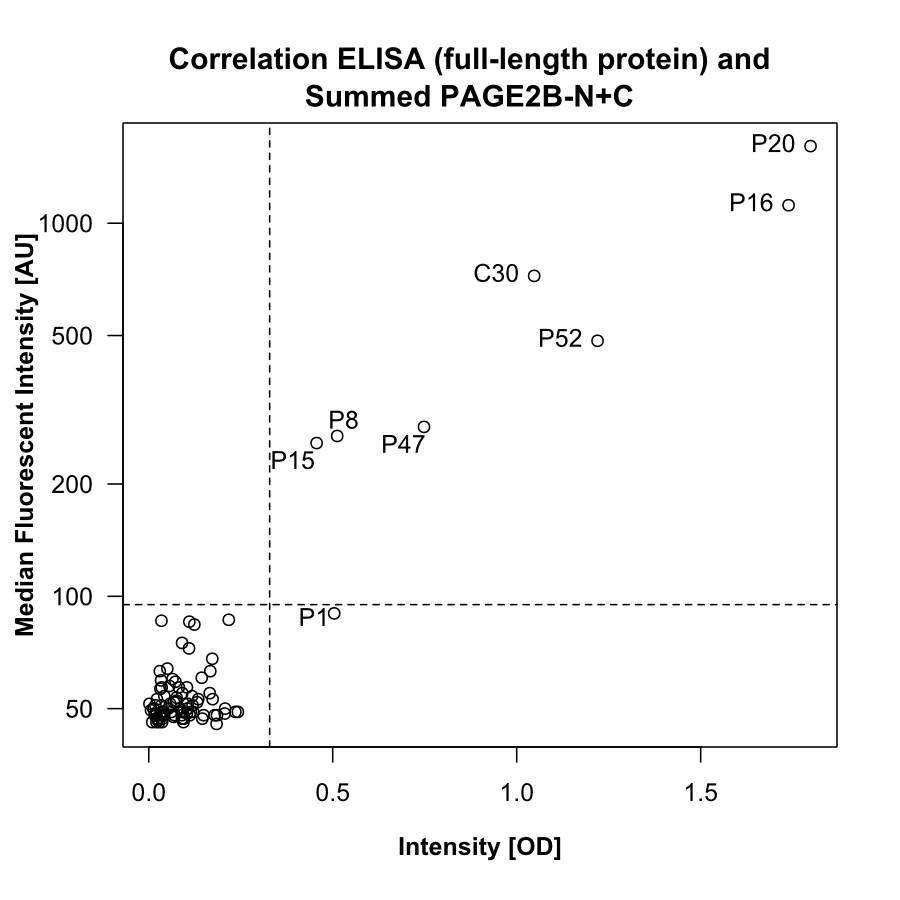

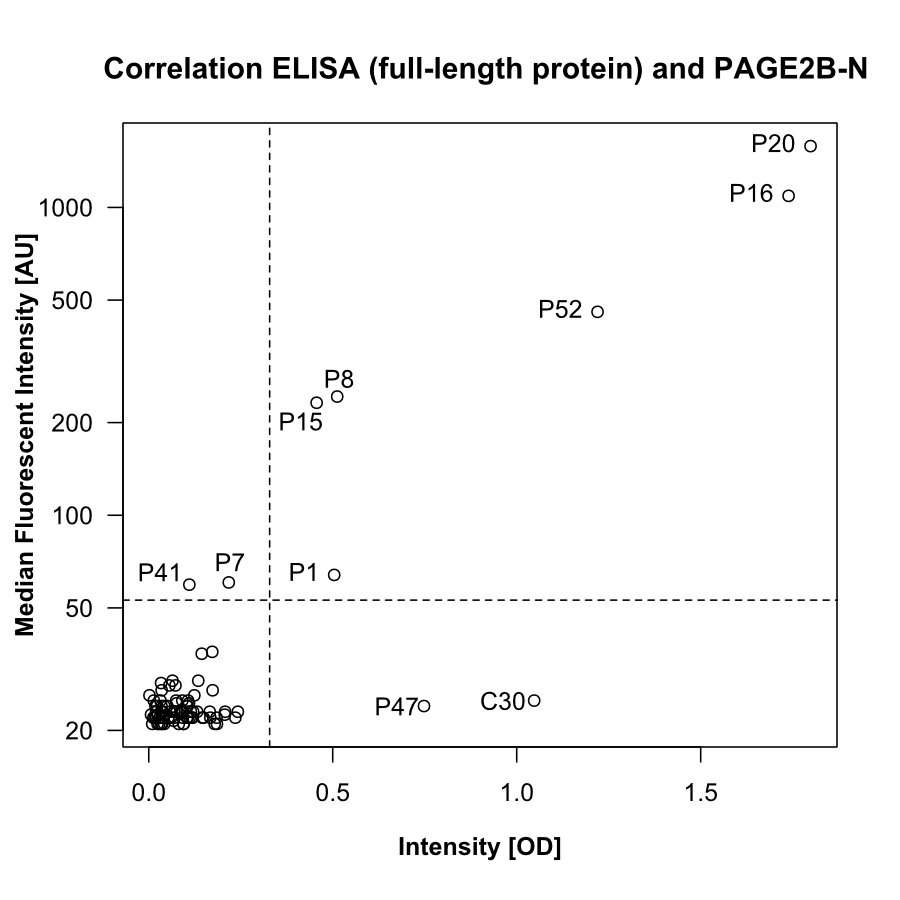

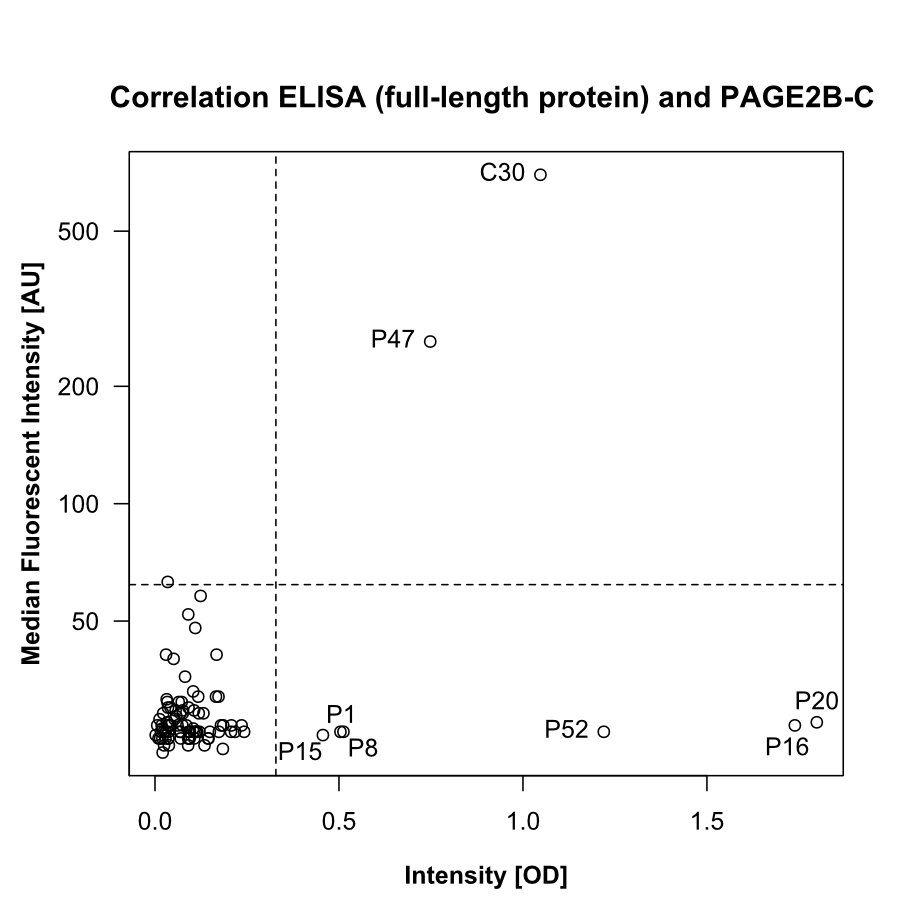


**a**

**b**

**c**

**Supplemental Methods**

**Epitope Mapping**

Epitope mapping of the plasma samples and rabbit antisera

Data from the epitope mapping from the suspension bead array were obtained from the FLEXMAP3D instrument (Luminex Corp.). The data, containing the signal intensities from the detection antibody were normalized sample-wise by subtracting each intensity value with the negative control, i.e. background levels given by the bare neutravidin bead. The peptides were sorted and consecutive peptides that showed signal above the background level were used to determine the epitopes, and the minimal epitopes by identifying the minimal number of aminoacids required for the autoantibody to bind.

Aligning sequence to other human proteins

Identified minimal epitopes were used to search for sequence similarity and potential cross reactivity to other human proteins by using BLASTP 2.2.29+^2^, using default settings and searching in the UniProtKB/Swiss-Prot database.

**References:**

1. Gjerstorff MF, Besir H, Larsen MR, Ditzel HJ. Expression, purification and characterization of the cancer-germline antigen GAGE12I: a candidate for cancer immunotherapy. *Protein Expr Purif* 2010; **73**(2)**:** 217-222.

2. Altschul SF, Madden TL, Schaffer AA, Zhang JH, Zhang Z, Miller W *et al.* Gapped BLAST and PSI-BLAST: a new generation of protein database search programs. *Nucleic Acids Res* 1997; **25**(17)**:** 3389-3402.
